# Hyperphosphatemia and risks of acute kidney injury, end-stage renal disease, and mortality in hospitalized patients

**DOI:** 10.1186/s12882-019-1556-y

**Published:** 2019-09-18

**Authors:** Hongran Moon, Ho Jun Chin, Ki Young Na, Kwon Wook Joo, Yon Su Kim, Sejoong Kim, Seung Seok Han

**Affiliations:** 1 Department of Internal Medicine, Seoul National University College of Medicine, 103 Daehakro, Jongno-gu, Seoul 03080 Korea; 2 Department of Internal Medicine, Seoul National University Bundang Hospital, 82, Gumi-ro 173beon-gil, Bundang-gu, Seongnam-si, Gyeonggi-do 13620 Korea

**Keywords:** Acute kidney injury, End-stage renal disease, Hyperphosphatemia, Mortality, Phosphorus

## Abstract

**Background:**

Hyperphosphatemia is associated with vascular calcification and bone mineral disorders and is a major concern among patients with chronic kidney disease (CKD). However, the relationship between hyperphosphatemia and renal outcome in non-CKD patients has not been studied. Furthermore, the clinical implications of hyperphosphatemia in relation to the risks of acute kidney injury (AKI), end-stage renal disease (ESRD), and mortality after hospitalization remain unresolved.

**Methods:**

A total of 20,686 patients (aged ≥18 years) admitted to Seoul National University Bundang Hospital from January 2013 to December 2013 were retrospectively reviewed. Patients were divided into quartiles according to serum phosphorus level at the time of admission. The odds ratios (ORs) for AKI and hazard ratios (HRs) for ESRD and all-cause mortality were calculated after adjustment of multiple covariates.

**Results:**

AKI developed in 2319 patients (11.2%), with higher ORs for patients in the third and fourth quartiles (1.4 [1.24–1.68] and 2.8 [2.44–3.22], respectively) compared with the first quartile group. During a median follow-up period of 4.0 years, 183 patients (0.88%) developed ESRD and 3675 patients (17.8%) died. Patients in the fourth quartile had higher risks of ESRD and mortality than patients in the first quartile (HRs, 2.3 [1.46–3.75] and 1.4 [1.22–1.49], respectively). These trends remained consistent in patients with an estimated glomerular filtration rate > 60 ml/min/1.73 m^2^.

**Conclusions:**

Hyperphosphatemia is related to the risks of AKI, ESRD, and mortality, and it may therefore be necessary to monitor serum phosphorus level in hospitalized patients, irrespective of kidney function.

## Background

Chronic kidney disease (CKD) is increasingly recognized as a public health problem [1] and is associated with higher risks of cardiovascular disease and other conditions, including infections and cancer [2]. Acute kidney injury (AKI) is a renal complication that occurs in up to 20% of hospital admissions [3, 4], and which can eventually progression to CKD. Both AKI and CKD thus represent significant health burdens with potentially high morbidity and mortality rates [5].

Serum phosphorus levels are regulated by multiple organs including the kidney, bone, and digestive systems [6]. Given that the kidney plays a major role in phosphorus regulation, phosphorus homeostasis can be disrupted in patients with kidney disease. Abnormal phosphorus levels may in turn be related to several morbidities, such as vascular calcification, mineral bone disease, and hyperparathyroidism [7, 8]. Furthermore, the risk of death has been shown to increase by approximately 20% for each 1 mg/dl increment in serum phosphorus among patients with CKD [9, 10]. Accordingly, the Kidney Disease Improving Global Outcomes (KDIGO) guidelines recommend monitoring serum phosphorus levels and lowering elevated levels towards the normal range in patients with CKD [11]. Hyperphosphatemia was also recently shown to increase the risk of cardiovascular disease in non-CKD patients [12, 13]. However, most studies of hyperphosphatemia have focused on certain disease subsets rather than considering all hospitalized patients. Furthermore, the relationship between hyperphosphatemia and AKI has not yet been fully established. The present study thus assessed the effects of hyperphosphatemia on the risks of AKI, ESRD, and mortality among a large number of hospitalized patients.

## Methods

### Study population

We retrospectively reviewed data for adult patients (aged ≥18 years) admitted to Seoul National University Bundang Hospital in 2013 (*n* = 21,572). Patients without measurements of serum phosphorus (*n* = 575) and/or with underlying ESRD (*n* = 311) were excluded. Consequently, a total of 20,686 patients were included in the present analysis.

### Data collection and definitions

The clinical parameters investigated were age, sex, body mass index, hypertension, diabetes mellitus, history of cardiovascular disease, CKD, and medications (including angiotensin-converting enzyme inhibitors or angiotensin receptor blockers, β-blockers, calcium channel blockers, and diuretics). Blood parameters including creatinine, hemoglobin, cholesterol, calcium, phosphorus, and albumin were also examined. Estimated glomerular filtration rate (eGFR) was calculated using the CKD Epidemiology Collaboration equation [14].

Primary outcomes were the occurrences of AKI and ESRD after hospital admission. AKI was defined according to the KDIGO guideline [15] as meeting at least one of the following criteria: increase in serum creatinine by 0.3 mg/dl within 48 h or increase to 1.5 times baseline, known or presumed to occur within the prior 7 days. Baseline creatinine was defined as the lowest value measured < 90 days prior to admission. If this value was unavailable, the lowest creatinine value measured between 90 and 180 days before admission was used as the baseline. The events of ESRD, such as dialysis and kidney transplantation, were obtained from the Kidney Renal Registry database of Korea. The additional patient outcome was all-cause mortality following admission. The mortality data were obtained from the national database of Statistics Korea. All patients, except patients who were death-censored, were followed up until July 2017.

### Statistical analysis

Statistical analyses were performed using SPSS (version 23.0; IBM Corp., Chicago, IL, USA) and STATA (version 12.0; Stata Corp LP, College Station, TX, USA) softwares. Continuous and categorical variables were expressed as the means ± standard deviations and the proportions, respectively. Categorical variables were compared by χ^2^ tests. Continuous variables were compared by an analysis of variance or a post-hoc analysis of least significant difference, based on the number of comparison groups. Non-linear relationships were evaluated using a restricted cubic spline analysis. Odds ratios (ORs) with 95% confidence intervals for the risk of AKI were calculated by a logistic regression, with and without adjustment for the covariates, such as age, sex, body mass index, hypertension, diabetes mellitus, history of cardiovascular disease, medications, eGFR, calcium, hemoglobin, cholesterol, and albumin levels. The relationships between serum phosphorus and ESRD or all-cause mortality were assessed using a Cox proportional hazard ratio (HR) model before and after adjustment for covariates (i.e., AKI plus the above covariates). The relationships were further analyzed after stratification according to baseline kidney function (i.e., eGFR < 60 ml/min/1.73 m^2^ or ≥ 60 ml/min/1.73 m^2^). *P* values < 0.05 were defined as significant when they were set to two-sided.

## Results

### Baseline characteristics

The baseline characteristics of the patients are shown in Table 1. The study included 20,686 patients (52.5% male), with a mean age of 59 years. Patients were split into quartiles according to serum phosphorus levels: first quartile, < 2.9 mg/dl; second quartile, 2.9–3.4 mg/dl; third quartile, 3.5–3.8 mg/dl; and fourth quartile, > 3.8 mg/dl. Patients in the first quartile were older at baseline and included more males compared with the other groups. More patients in the fourth quartile were diagnosed with hypertension, but there was no difference in history of cardiovascular disease among the groups. Patients in the lower quartiles tended to have lower cholesterol and higher eGFR levels. The mean follow-up period was 3.6 ± 1.2 years.

**Table 1:** Baseline characteristics of study participants

		Serum phosphorus level				
Variables	Total (*n* = 20,686)	1st quartile (*n* = 5660)	2nd quartile (*n* = 5717)	3rd quartile (*n* = 4487)	4th quartile (*n* = 4822)	*P*
Age (years)	58.9 ± 17.0	61.4 ± 16.1	59.5 ± 16.7^‡^	57.6 ± 17.1^‡^	56.5 ± 18.1^‡^	< 0.001
Male sex (%)	52.5	57.4	55.2	50.6^‡^	46.1^‡^	< 0.001
Body mass index (kg/m^2^)	23.7 ± 3.7	23.6 ± 3.7	23.8 ± 3.6	23.7 ± 3.6^*^	23.9 ± 3.9^†^	0.002
Comorbidities (%)						
Hypertension	19.0	17.9	18.6	18.4	21.5^‡^	< 0.001
Diabetes mellitus	21.9	22.5	20.8	19.7^†^	24.5	< 0.001
History of cardiovascular disease	6.1	5.8	6.3	6.7	5.8	0.164
Chronic kidney disease	8.0	8.7	7.0^*^	7.0^*^	9.4	< 0.001
Medications (%)						
ACEi	1.4	1.1	1.3	1.6	1.6	0.180
ARB	6.4	5.9	6.2	6.4	7.2^*^	0.040
Beta blocker	5.9	4.5	6.1^†^	6.2^†^	7.1^‡^	< 0.001
Calcium channel blocker	7.1	6.7	6.9	7.2	7.7^*^	0.193
Diuretics	5.3	5.2	4.9	4.6	6.4^†^	0.001
Laboratory findings						
Hemoglobin (g/dL)	12.6 ± 2.1	12.3 ± 2.2	12.8 ± 2.0^‡^	12.9 ± 2.0^‡^	12.7 ± 2.2^‡^	< 0.001
Albumin (g/dL)	3.9 ± 0.6	3.7 ± 0.6	4.0 ± 0.6^‡^	4.0 ± 0.5^‡^	4.0 ± 0.6^‡^	< 0.001
Total cholesterol (mg/dL)	170.1 ± 47.5	157.3 ± 46.4	171.2 ± 43.8^‡^	177.5 ± 47.3^‡^	177.2 ± 50.0^‡^	< 0.001
Calcium (mg/dL)	8.6 ± 0.7	8.3 ± 0.7	8.7 ± 0.6^‡^	8.8 ± 0.5^‡^	8.8 ± 0.7^‡^	< 0.001
Phosphorus (mg/dL)	3.38 ± 0.81	2.46 ± 0.41^‡^	3.21 ± 0.14^‡^	3.64 ± 0.11^‡^	4.38 ± 0.73^‡^	< 0.001
Creatinine (mg/dL)	0.82 ± 0.72	0.77 ± 0.42	0.76 ± 0.57	0.77 ± 0.44	1.00 ± 1.28^‡^	< 0.001
eGFR (mL/min/1.73 m^2^)	86.0 ± 35.5	88.9 ± 37.4	85.6 ± 36.8^‡^	84.6 ± 30.2^‡^	84.4 ± 36.2^‡^	< 0.001

Comparisons were evaluated using the Kruskal wallis test for categorical variables and analysis of variance for continuous variables (post-hoc analysis of least significant difference between the two groups). The first quartile group served as a reference for the comparison between the two groups

*ACEi* angiotensin-converting enzyme inhibitor, *ARB* angiotensin II receptor blocker, *eGFR* estimated glomerular filtration rate

*P <  0.05; ^†^*P* <  0.01; ^‡^*P* <  0.001, compared with the 1st quartile

### Association between serum phosphorus and renal outcome

**Fig. 1:**
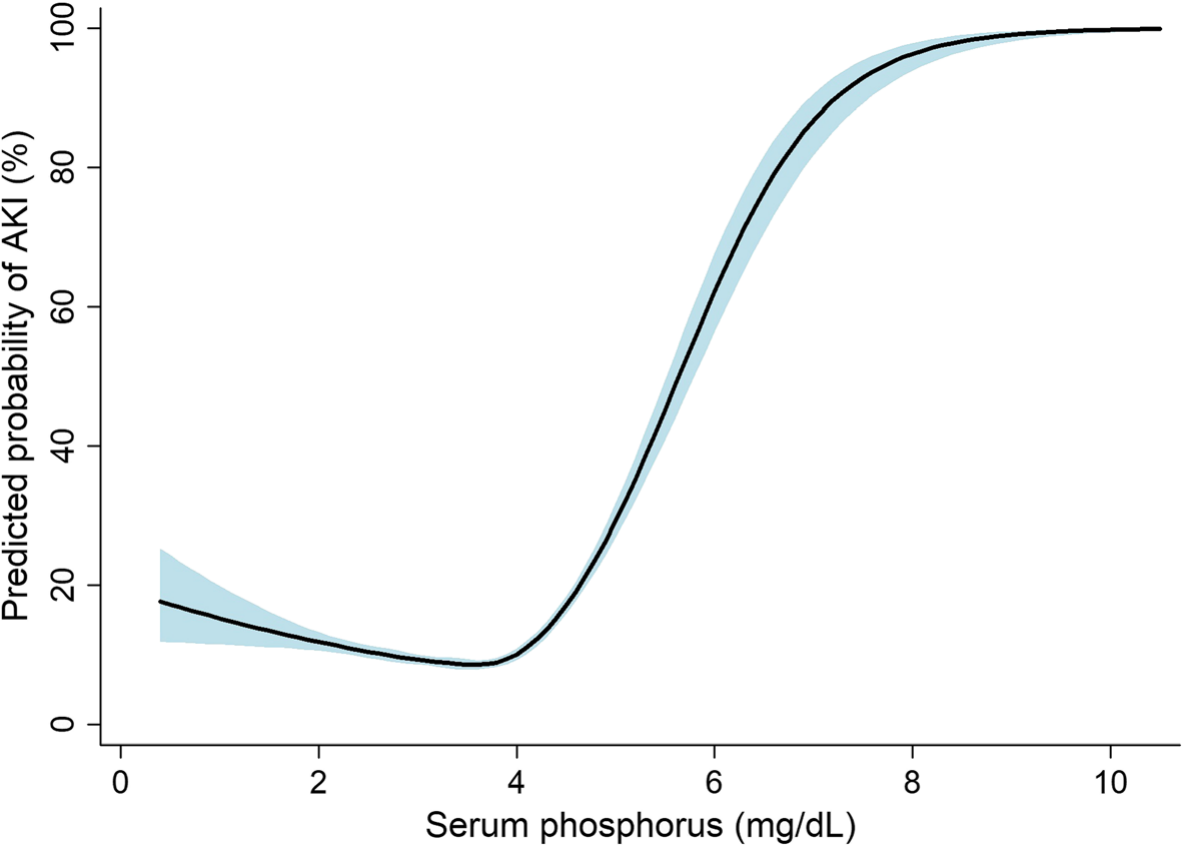
Non-linear relationship between serum phosphorus and predicted probability of acute kidney injury (AKI). Fitted line and 95% confidence intervals indicated as solid and shaded areas, respectively

AKI occurred in 2321 (11.2%) patients after hospital admission, with prevalence of 10.6, 8.8, 9.2, and 16.6% in the first to fourth quartiles, respectively. Non-linear relationship analysis demonstrated an exponential relationship between serum phosphorus and the risk of AKI (Fig. 1). Logistic regression analysis showed a U-shaped relationship between phosphate and AKI (ORs: 0.82 [0.720–0.924] in the second quartile, *P =* 0.001; 0.85 [0.744–0.969] in the third quartile, *P* = 0.015; 1.68 [1.499–1.881] in the fourth quartile, *P* <  0.001). The positive relationship between phosphate level and risk of AKI remained consistent after adjusting for multiple covariates (Table 2), indicating that the trend was not dependent on reduced kidney function.

**Table 2:** Risk of acute kidney injury according to serum phosphorus level

	Total		eGFR < 60 ml/min/1.73 m^2^		eGFR ≥60 ml/min/1.73 m^2^	
Groups	OR (95% CI)	*P*	OR (95% CI)	*P*	OR (95% CI)	*P*
Q1	1 (reference)		1 (reference)		1 (reference)	
Q2	1.16 (1.004–1.340)	0.044	0.99 (0.657–1.480)	0.946	1.18 (1.012–1.383)	0.034
Q3	1.44 (1.237–1.684)	< 0.001	1.38 (0.916–2.077)	0.124	1.41 (1.194–1.673)	< 0.001
Q4	2.80 (2.435–3.222)	< 0.001	2.64 (1.843–3.770)	< 0.001	2.60 (2.226–3.038)	< 0.001

*eGFR* estimated glomerular filtration rate, *OR* odds ratio, *CI* confidence interval

**Fig. 2:**
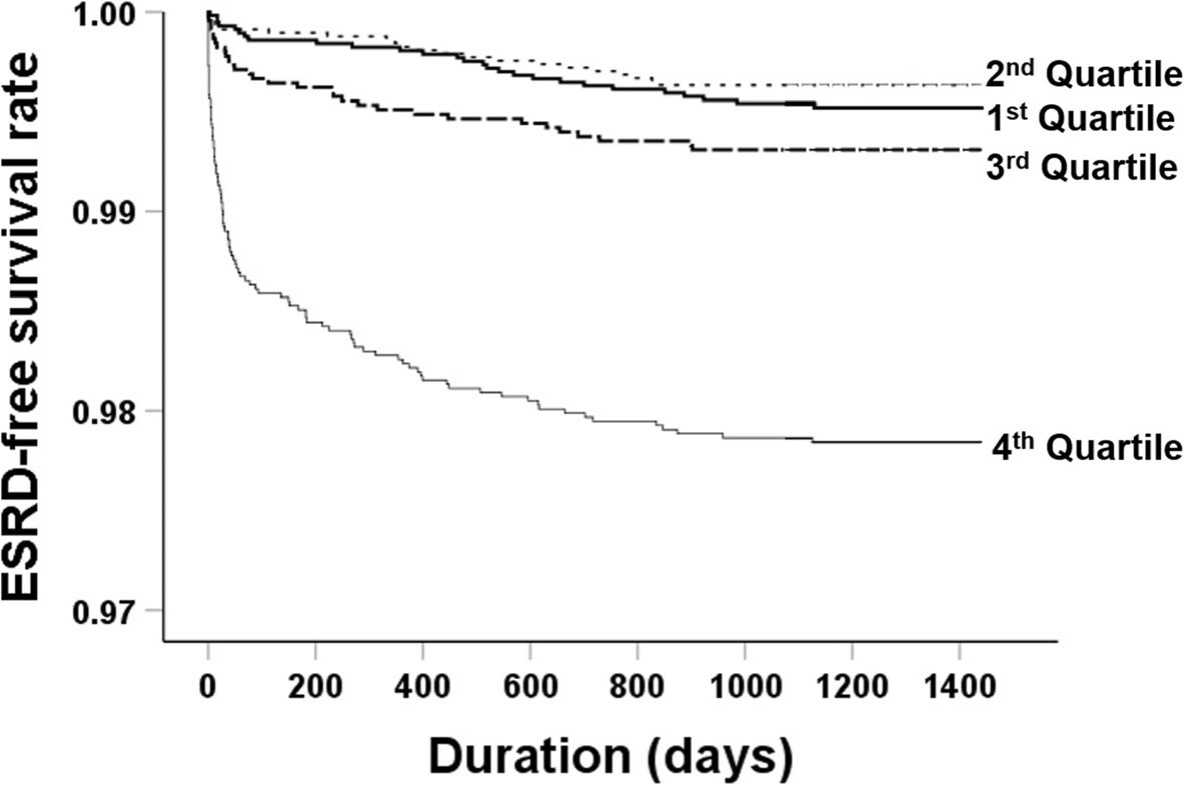
Kaplan–Meier curves for end-stage renal disease (ESRD) according to quartiles of serum phosphorus

Sixty-three patients (0.3%) progressed to ESRD status based on kidney function during the follow-up period. The prevalence of ESRD were 0.2, 0.1, 0.3, and 0.8% in the first to fourth quartile groups, respectively. Kaplan–Meier curves for ESRD-free survival rates indicated the highest risk of ESRD in the fourth quartile group (Fig. 2). The HR for the risk of ESRD was 2.34 (1.462–3.750) in the fourth quartile group compared with the first quartile group (Table 3). The HR for ESRD remained higher in the fourth compared with the first quartile group after adjusting for multiple covariates, but this trend was only significant in patients with an eGFR > 60 ml/min/1.73 m^2^.

**Table 3:** Risk of end-stage renal disease according to serum phosphorus level

	Total		eGFR < 60 ml/min/1.73 m^2^		eGFR ≥60 ml/min/1.73 m^2^	
Groups	HR (95% CI)	*P*	HR (95% CI)	*P*	HR (95% CI)	*P*
Q1	1 (reference)		1 (reference)		1 (reference)	
Q2	0.95 (0.609–2.077)	0.871	0.99 (0.464–2.095)	0.971	0.94 (0.323–2.736)	0.910
Q3	1.45 (1.342–4.015)	0.185	0.83 (0.399–1.712)	0.608	2.74 (1.110–6.748)	0.029
Q4	2.34 (1.462–3.750)	< 0.001	1.17 (0.654–2.100)	0.595	5.25 (2.361–11.659)	< 0.001

*eGFR* estimated glomerular filtration rate, *HR* hazard ratio, *CI* confidence interval

As a sensitivity analysis, we divided patients into three groups such as low (< 2.5 mg/dl), normal (2.5–4.5 mg/dl), and high phosphorus (> 4.5 mg/dl) groups, based on the reference range. Nevertheless, the risks of AKI and ESRD were higher in high phosphorus group than in the normal group, wherein adjusted OR and HR were 6.12 (5.187–7.209) (*P* <  0.001) and 1.91 (1.311–2.777) (*P* = 0.001), respectively. The low group was not associated with the risk of AKI or ESRD (*P*s > 0.05).

### Association between phosphorus and mortality

**Fig. 3:**
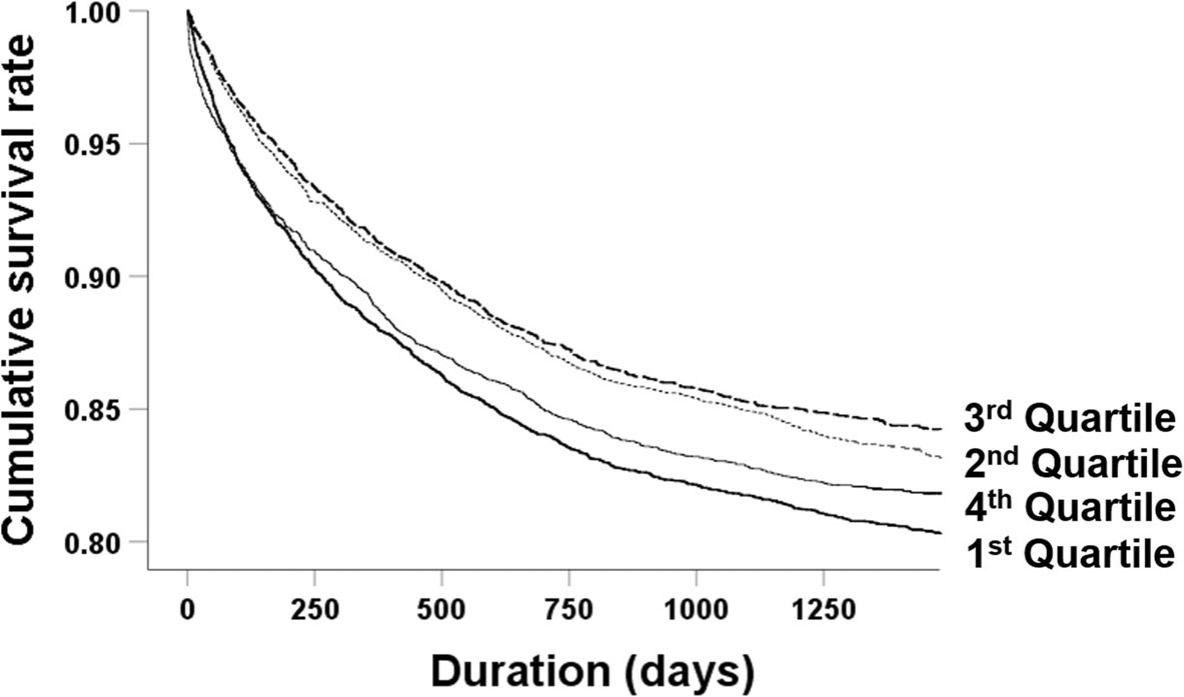
Kaplan–Meier curves for all-cause mortality according to quartiles of serum phosphorus

The all-cause mortality rates were 19.8, 16.9, 15.7, and 18.3% in the first to fourth quartile groups, respectively, and the Kaplan–Meier survival curves showed similar trends (Fig. 3). Univariate analysis showed that the HRs for mortality were lower in the second and third quartile groups (0.83 [0.761–0.904], *P* <  0.001 and 0.77 [0.702–0.848], *P* <  0.001, respectively) compared with the first quartile group. The mortality rates did not differ between the first and fourth quartile groups. However, the mortality risk tended to be higher in the higher quartiles compared with the first quartile group after adjusting for multiple covariates (Table 4). Subgroup analysis revealed positive correlations between serum phosphorus level and risk of mortality in both CKD and non-CKD groups. Although a sensitivity analysis was performed, the high phosphorus group, and not the low phosphorus group, had a higher risk of mortality than the normal group with adjusted HR of 1.54 (1.357–1.755) (*P* <  0.001).

**Table 4:** Risk of all-cause mortality according to serum phosphorus level

	Total		eGFR < 60 ml/min/1.73 m^2^		eGFR ≥60 ml/min/1.73 m^2^	
Groups	HR (95% CI)	*P*	HR (95% CI)	*P*	HR (95% CI)	*P*
Q1	1 (reference)		1 (reference)		1 (reference)	
Q2	1.10 (1.002–1.207)	0.052	1.00 (0.761–1.309)	0.987	1.11 (1.008–1.230)	0.034
Q3	1.16 (1.043–1.281)	0.006	1.28 (0.976–1.691)	0.075	1.14 (1.019–1.272)	0.022
Q4	1.35 (1.221–1.486)	< 0.001	1.37 (1.064–1.770)	0.015	1.34 (1.199–1.487)	< 0.001

*eGFR* estimated glomerular filtration rate, *HR* hazard ratio, *CI* confidence interval

## Discussion

Hyperphosphatemia has received considerable attention in the field of CKD - mineral and bone disorder (CKD-MBD) because of its association with cardiovascular morbidity and mortality [3, 6, 16]. Even upper-normal serum phosphate levels could exacerbate the status of CKD, potentially leading to ESRD [17]. Based on these features, this study addressed the relationships between hyperphosphatemia and the risks of AKI, ESRD, and mortality among hospitalized patients, and showed that hyperphosphatemia was significantly associated to poorer renal outcomes, irrespective of baseline kidney function.

In addition to patients with CKD-MBD, some studies have examined the relationship between serum phosphorus and morbidity or mortality, particularly in critically ill patients. Patients admitted to the emergency department with serum phosphorus levels > 4.5 mg/dl showed high 28-day in-hospital mortality [18]. Furthermore, higher time-weighted phosphorus levels (i.e., area under the curve divided by total time between first and last phosphorus levels) were related to mortality risk in patients with mechanical ventilation [19]. Hyperphosphatemia was also shown to affect the risk of mortality in patients undergoing continuous renal replacement therapy [20]. However, the relevance of hyperphosphatemia among heterogeneous patients admitted to the hospital remains unclear. Furthermore, AKI and ESRD have rarely been considered as the primary outcomes in previous studies [21].

In the current study, we examined the relationships between hyperphosphatemia and renal outcomes and mortality in both eGFR < 60 ml/min/1.73 m^2^ and ≥ 60 ml/min/1.73 m^2^ groups, to highlight the dangers of high phosphorus levels irrespective of kidney function. Hyperphosphatemia induced calcification of smooth muscle cells in an in vitro system [22], linked to phenotypic changes from vascular smooth muscle cells to osteochondrogenic-like cells [22]. Vascular calcification suggests an increased risk of cardiovascular events and mortality in patients with hyperphosphatemia. Some studies also found that high phosphate intake disrupted endothelial function and thus affected maintenance of the glomerular filtration barrier [17–19, 23–25]. This impaired vasodilatation is known to be dependent on nitric oxide [18, 25]. The Ramipril Efficacy In Nephropathy (REIN) study conducted in patients with non-diabetic chronic nephropathies found that a 1 mg/dl increase in serum phosphate level was associated with a 85% excess risk of progression to ESRD [25, 26], possibly associated with high secretion of fibroblast growth factor 23 [25]. Following docking of fibroblast growth factor, the respective receptor increases the production of angiotensin-converting enzyme, which may in turn activate the renin–angiotensin system, potentially facilitating the onset and progression of kidney damage in high-risk individuals [26]. Hyperphosphatemia also induced inflammation in vascular smooth muscle cells and increased the expression of matrix metalloproteinases II and IX and cathepsin S [9, 27]. These mechanisms may explain the finding that high concentrations of phosphorus affected renal outcomes, as well as mortality.

The strengths of this study included the large number of hospitalized patients analyzed and its lack of focus on critically ill patients or patients with CKD alone. Nevertheless, the study had some limitations. The retrospective design meant that the causality and mechanism could not be determined. However, this did not affect the primary purpose of the study to determine the relationship between serum phosphorus and renal outcomes. Unidentified factors may also have affected or interacted with the present relationships. We did not investigate the cause of death, which also limited our understanding of the underlying mechanisms. Because of relatively small number of patients with CKD, further studies are needed to assess non-significant results such as ESRD progression in CKD subset.

## Conclusion

Hyperphosphatemia is associated to the risks of AKI, ESRD, and mortality among hospitalized patients. Accordingly, serum phosphorus monitoring may help to identify patients at high risk of kidney function deterioration or mortality. Further studies are needed to explore the mechanisms underlying the present observations.

## Data Availability

The datasets used and analyzed during the current study are available from the corresponding author on reasonable request.

## Abbreviations

ACEi: Angiotensin-converting enzyme inhibitor
AKI: Acute kidney injury
ARB: Angiotensin II receptor blocker
CKD: Chronic kidney disease
CKD-MBD: CKD-mineral and bone disorder
eGFR: Estimated glomerular filtration rate
ESRD: End-stage renal disease
HR: Hazard ratios
OR: Odds ratios
